# Functional Modular Network Identifies the Key Genes of Preoperative Inhalation Anesthesia and Intravenous Anesthesia in Off-Pump Coronary Artery Bypass Grafting

**DOI:** 10.1155/2020/4574792

**Published:** 2020-08-17

**Authors:** Hongfei Zhao, Weitian Wang, Liping Liu, Junlong Wang, Quanzhang Yan

**Affiliations:** ^1^Department of Anesthesiology, Weifang People's Hospital, Weifang 261000, China; ^2^Department of Neurology, Weifang People's Hospital, Weifang 261000, China

## Abstract

Off-pump coronary artery bypass grafting (OPCABG) is an effective strategy for revascularization. Preoperative anesthesia appears critical due to surgical instability and the risk of organ damage. This study, based on a functional module network, analysed the effects of preoperative inhalation anesthesia and intravenous anesthesia on OPCABG and performed a pivot analysis of its potential drug regulators. We obtained microarray data of sevoflurane anesthesia and propofol anesthesia from the GEO database and analysed the difference between the two groups of data, resulting in 5701 and 3210 differential genes to construct the expression matrix. WGCNA analysis showed that sevoflurane anesthesia clustered into 7 functional disorder modules, including PDCD6IP, WDR3, and other core genes; propofol anesthesia clustered to form two functional disorder modules, including KCNB2 and LHX2, two core genes Enrichment analysis of the functions and pathways of interest suggests that both anesthesia-related module genes tend to function as pathways associated with ion and transmembrane transport. The underlying mechanism may be that targeted regulation of transmembrane-associated biological processes and ion pathways in the core genes of each module affect the surgical process. Pivot analysis of potential drug regulators revealed 229 potential drugs for sevoflurane anesthesia surgery, among which zinc regulates three functional disorder modules via AHSG, F12, etc., and 67 potential drugs for propofol anesthesia surgery, among which are propofol, methadone, and buprenorphine, regulate two functional disorder modules through four genes, CYP2C8, OPRM1, CYP2C18, and CYP2C19. This study provides guidance on clinical use or treatment by comparing the effects of two anesthesias on surgery and its potential drugs.

## 1. Introduction

Currently, off-pump coronary artery bypass grafting (OPCABG) is an innovative technique in cardiac surgery. In recent years, an aging population, increased risk of surgery, and improved technology contribute to the resurgence of OPCABG [1]. OPCABG can reduce postoperative complications such as systemic inflammatory response, myocardial damage, kidney damage, and brain damage [2]. Currently, it is the best choice for modern cardiac surgery. Mortality and stroke rate of the elderly after surgery are extremely low, indicating that surgery is the safe management option for coronary artery disease in this population [3]. However, during the operation, the patient needs to be anesthetized. Thus, choosing which kind of anesthesia is very important. Common methods are inhalation anesthesia, intravenous anesthesia, sevoflurane anesthesia, and propofol anesthesia. Compared with propofol-based total intravenous anesthesia (TIVA), sevoflurane anesthesia reduced cardiac biomarker release and hospital stay. It also could reduce mortality compared with CFG [4]. This may be due to the fact that sevoflurane can better protect the heart muscle during cardiac surgery [5]. The induction characteristics of sevoflurane anesthesia in congenital heart disease are similar. Sevoflurane induced good tolerance technology, suitable for children with congenital heart disease [6]. Although sevoflurane anesthesia has numerous benefits, when sevoflurane is paused to use after surgery, rhythmic heart separation returns to sinus rhythm. Sometimes, it might cause atrioventricular conduction disturbances, leading to rhythmic arrhythmias [7]. Another common anesthetic is propofol. It can be quickly induced and rapidly eliminated with short duration of action, smooth recovery of anesthesia, and few side effects. So it has been widely used. It is more effective and harmless than hypnotic drug [8]. Propofol is also considered as the best anesthetic alternative in experiments comparing the recovery periods of the two anesthesia regimens [9]. Propofol is also reported to be approved for continuous intravenous sedation. Surgical clinical studies have revealed that the combination of propofol and opioids is a reasonable anesthetic option [10]. OPCABG surgery is associated with lymphopenia. Propofol anesthesia with protective effects is superior to sevoflurane maintenance anesthesia [11]. When it comes to the difference between the two anesthesia methods, the researchers have many different opinions. Of course, some people thought that no difference exists in myocardial protection after sevoflurane anesthesia or propofol anesthesia in OPCABG surgery [12]. Although propofol-induced anesthesia can attenuate the feedback pathway of cardiac baroreflex and the feedforward pathway can be immune to anesthesia [13], we still have no conclusion about the use of the two anesthetic methods. To further investigate the difference between the two anesthetic methods, we conducted a study on molecular mechanisms of its regulation.

Here, we analysed the effects of postoperative inhalation anesthesia and intravenous anesthesia on off-pump coronary artery bypass grafting based on a functional modular network, to explore the underlying molecular mechanisms.

## 2. Materials and Methods

### 2.1. Differential Expression Analysis

We collected an expression microarray dataset for inhaled anesthesia and intravenous anesthesia prior to coronary artery bypass grafting from the NCBI Gene Expression Omnibus (GEO) database [14], numbered GSE4386, and performed variance analysis in the collected disease samples (containing interleukin 23; no interleukin 23) using the R language limma package [15]. With threshold *P* < 0.05, significantly differentially expressed genes were obtained. In the end, a total of 6699 differential genes were obtained, including 2212 common genes, 3489 specific differential genes for sevoflurane anesthesia, and 998 differentially expressed genes for propofol anesthesia. We used two sets of differential genes to construct an expression profile matrix for nonexternal coronary artery bypass grafting.

### 2.2. Coexpression Analysis

WGCNA [16] on two sets of differential expression profiles, respectively, clusters similar or identical genes to form a module, also known as a functional disorder module, in order to explore synergistically express relationships of differential genes in the two groups. Since the two sets of data in this study are subject to scale-free networks, correlation coefficients can be used for cluster analysis. Firstly, the correlation coefficient between the genes is taken to the *N*^th^ power by the correlation coefficient weighting, and the Person Coefficient between the genes is obtained. Then, the results of the Person Coefficient are clustered to obtain the clustering tree. Various branches of the cluster tree means various functional barrier modules while diverse colours represent diverse modules, and the genes of the same branch have strong correlations. There are numerous regulatory genes in each module, and we have extracted these genes with relatively large regulatory powers as key genes leading to the dysfunction of functional modules. Seven key genes were obtained for sevoflurane anesthesia-related modules, namely PDCD6IP, DNAH10, WDR3, PROP1, ASCL2, LRRC2-AS1, and SDC3. In addition, the two key genes of the module related to propofol anesthesia are KCNB2 and LHX2, respectively. Therefore, we believe that these core genes are involved in the molecular regulation of anesthesia for nonexternal coronary artery bypass grafting.

### 2.3. Analysis of Functional and Pathway Enrichment

Exploring the function and signalling pathways involving genes is often an effective means of studying the molecular mechanisms of disease. We performed the GO function and KEGG pathway enrichment analysis for the differentially expressed genes of seven functional disorders related to sevoflurane anesthesia and two functional disorder modules related to propofol anesthesia. The enrichment analysis of the GO function and the KEGG pathway uses the clusterProfiler package in the R language [17, 18], with threshold pvalueCutoff = 0.05. Through the perspective of data, we filter out the functions and paths of interest that interact with multiple modules and draw bubble maps based on the count values that act between the modules. This study screened 15 interesting functions and pathways, and used ggplot2 in R language to draw bubble maps for display. The size of the bubble in the display represents the count value of the function and the path. The larger the value of the count, the more obvious the potential effect, while the colour refers to its LogFC value.

### 2.4. Identification of Drug Regulation of Modular Genes

To further enhance the impact of the two anesthetics on surgery, we performed a pharmacomodulator predictive analysis of the functional modules. Comparing the effects of two different anesthesia methods on the operation and the mechanism of action leaves guidance for clinical application. A pivotal analysis of the effects of the drug supplements given during anesthesia on the surgery will provide a better understanding of the effects of the drug on anesthesia. We calculated the enrichment target significance in each module based on the hypergeometric test (pvalueCutoff = 0.01, LogFC = 0.5) and obtained the drug related to the module. The data was input into Cytoscape for a module-drug network diagram. 229 drugs with potential effects on sevoflurane anesthesia were obtained. We selected related drugs and modules for network regulation. In addition, there are 67 drugs with potential effects on the operation of propofol anesthesia. We have a network diagram of potential interactions between drugs in the module for the interaction between these 67 modules and drugs.

### 2.5. Potential Role of Drug Target Genes

After analysing the interaction between the module and the drug, we do not know about the regulated target gene, so we need to analyse its target gene. This study was based on the pivot analysis of drugs and modules and review of the target gene for drug action in the DrugBank database, with threshold *P* < 0.05. We interpreted drugs with potential effect on sevoflurane anesthesia and propofol anesthesia as well as the relationship list of their target genes to Cytoscape and got regulatory network diagram of Module_Drug_TargetGene. When constructing the Module_Drug_TargetGene regulatory network map for the interaction of sevoflurane anesthesia surgery, we selected the zinc-related interaction relationship mapping.

## 3. Results

### 3.1. Time Series Expression of Dysregulated Molecules for Postoperative Anesthesia for Off-Pump Coronary Artery Bypass Grafting

**Figure 1:**
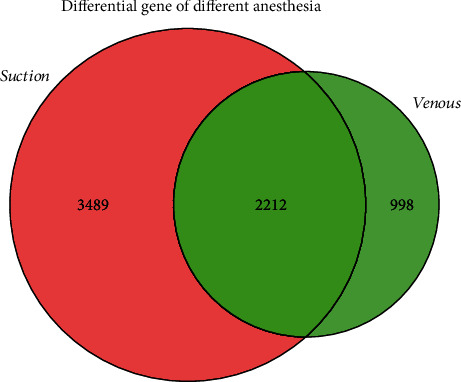
Venn diagram: Red represents the differential gene of sevoflurane anesthesia surgery, green represents the differential gene of propofol anesthesia, and the middle overlap is the difference gene shared by both.

First, we constructed the gene expression profiles of OPCABG under two anesthesia and analysed the differences in order to further understand the effect of anesthesia on off-pump coronary artery bypass grafting. Resulting in 5701 differential genes from sevoflurane anesthesia CABG surgery and 3210 differential genes from propofol anesthesia CABG surgery. The Venn map of the two groups of differential genes revealed 6699 differential genes comprising 2212 shared genes, 3489 specific for sevoflurane anesthesia, and 998 specific for propofol anesthesia (Figure 1).

### 3.2. Identification of Functional Module Networks

**Figure 2:**
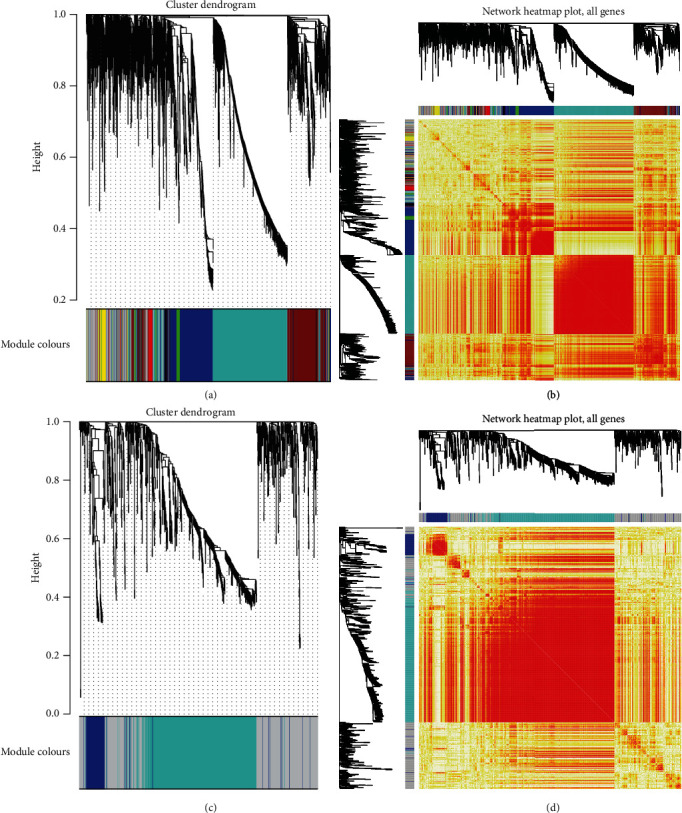
Synergistic expression of preoperative anesthesia OPCAB. (a) Synergistic expression of sevoflurane anesthesia for OPCABG, 7 coexpression groups obtained by clustering were identified as modules, and 7 colours represented 7 coexpression modules. (b) Here are heatmaps of all genes expression in the sample, whose expression behaviour is clustered into 7 coexpression modules. (c) Coexpression of propofol anesthesia for OPCABG, two coexpression groups obtained by clustering were identified as modules, and two colours represented two coexpression modules. (d) Propofol anesthetizes the expression heatmap of all genes in the sample, and its expression behaviour is clustered into two coexpression modules.

To further explain the effect of two anesthesia methods on off-pump coronary artery bypass grafting, based on the WGCNA analysis, we constructed 7 functional disorder modules using sevoflurane anesthesia-specific differential genes (Figures 2(a) and 2(b)). Two functional disorder modules were constructed by specific differential genes of propofol anesthesia (Figures 2(c) and 2(d)). In addition, we identified the module's hub genes by regulation of genes within the functional disorder module (Tables 1 and 2). Then, we analysed the various effect of its key genes in two anesthesia situations that participated in different functions and pathways on OPCABG.

### 3.3. Functions and Pathways Involved in the Gene of Interest

**Figure 3:**
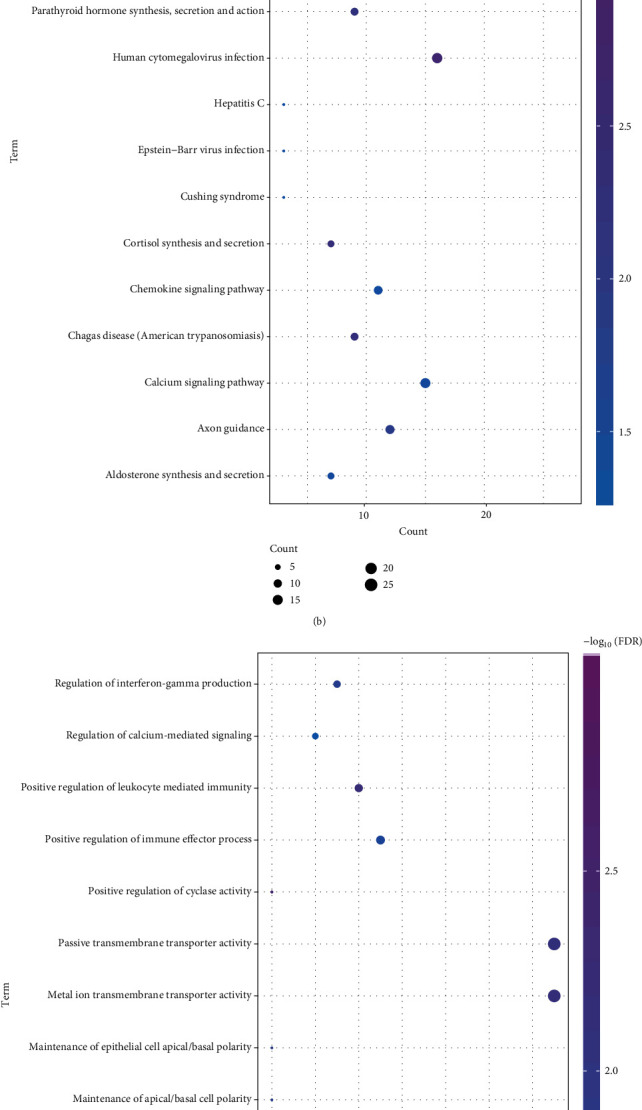
Functional and pathway enrichment analysis excerpts of preoperative anesthesia for OPCABG. (a) Sevoflurane anesthesia for excision analysis of GO gene functional enrichment of OPCAB. From blue to purple, the enrichment increases significantly. The larger the circle, the greater the proportion of the module gene in the GO function entry gene. (b) Sevoflurane anesthesia for excision analysis of the KEGG gene functional enrichment of OPCABG. From blue to purple, the enrichment increases significantly. The larger the circle, the greater the proportion of the module gene in KEGG function entry gene. (c) Propofol anesthesia for excision analysis of the GO gene functional enrichment of OPCABG. From blue to purple, the enrichment increases significantly. The larger the circle, the greater the proportion of the module gene in GO function entry gene. (d) Propofol anesthesia for OPCABG modular gene KEGG pathway enrichment analysis excerpt. From blue to purple, the enrichment increases significantly. The larger the circle, the greater the proportion of the module gene in KEGG function entry gene.

In order to further understand the biological characteristics of the functional impairment module, we performed the GO function and KEGG pathway enrichment analysis for the two functional module networks. The sevoflurane anesthesia-related module gene was involved in 371 cell composition entries, 680 molecular function terms, 2567 biological processes, and 128 signal pathways (Figures 3(a) and 3(b)). In light of functional analysis, we observed that related functional modules favour various biological process-related functions, including axonogenesis, anion antiporter activity, regulation of neurotransmitter levels, and PI3K-Akt signalling pathway. Besides, propofol anesthesia-related module genes involved 84 cell component entries, 179 molecular functional terms, 648 biological processes, and 15 signalling pathways (Schedule 2-2, Figures 3(c) and 3(d)). We observed that the relevant functional modules are mainly involved in ion channel and transmembrane transport, such as metal ion transmembrane transporter activity, channel activity, passive transmembrane transporter activity, and neuroactive ligand-receptor interaction. These signalling pathways have been shown to be associated with the development and progression of OPCABG under anesthesia.

### 3.4. Drugs with Potential Effects on OPCABG under Anesthesia

**Figure 4:**
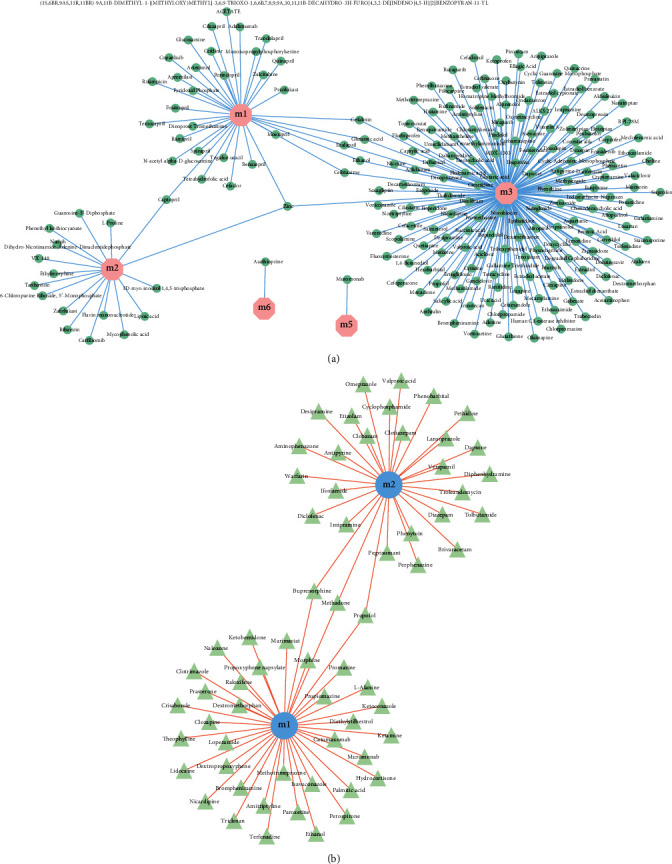
Regulation of drugs on dysfunction modules. (a) Potential module_drug regulatory relationship for preoperative sevoflurane anesthesia, with pink octagons representing modules and blue-green circles representing potential drugs. (b) Potential module_drug regulation of propofol anesthesia before surgery, with blue circles representing modules and green triangles representing potential drugs.

A pharmacomodulator predictive analysis of the functional modular genes was carried out for the impact of the two anesthetics on surgery. According to the number of regulatory modules with *P* value < 0.05, 229 drugs with potential effects on sevoflurane anesthesia were obtained (Schedule 3-1, Figure 4(a)), in which zinc significantly participated in the regulation of three modules. In addition, 67 drugs with potential effects on propofol anesthesia surgery (Schedule 3-2, Figure 4(b)), among which are propofol, methadone, and buprenorphine, significantly involved in the regulation of the two modules. Most drugs can have certain auxiliary or obstructive effects on anesthesia with certain impact on the surgical procedure.

### 3.5. Potential Role of Drug Target Genes

**Figure 5:**
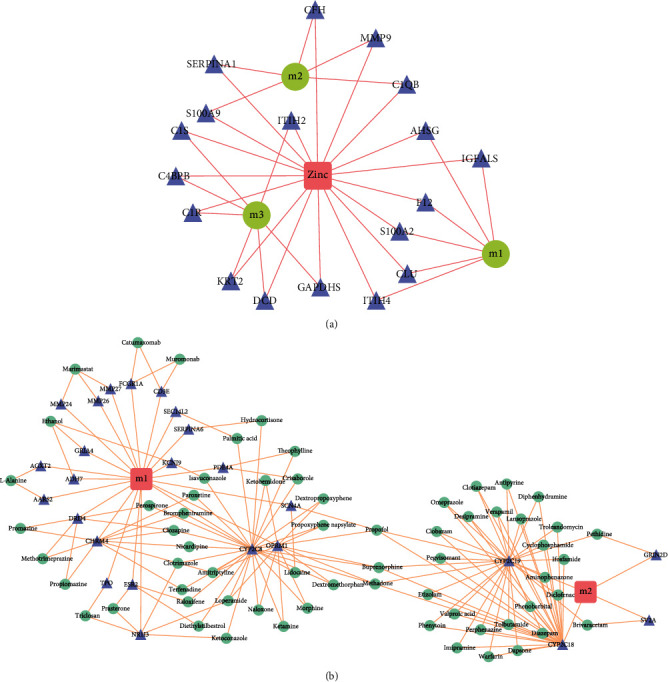
Regulation of drug target genes on dysfunction modules. (a) Potential Module_Drug_TargetGene regulation map for preoperative sevoflurane anesthesia, with green circles representing modules, pink quadrilateral for drugs, and purple triangles for target genes. (b) Potential Module_Drug_TargetGene regulation map of propofol anesthesia before surgery, pink quadrilateral represents the module, blue-green circles represent drugs, and purple triangles represent target genes.

Based on the pivot analysis of the drug, the target gene for drug action was traced back through the DrugBank database with threshold *P* < 0.05. Module_Drug_TargetGene regulatory relationship table for its target genes and drugs with potential effects on sevoflurane anesthesia was obtained, and zinc-related interactions were selected to construct a regulatory network map (Figure 5(a)). The figure indicates that zinc affects the progression of OPCABG through AHSG, F12, and other genes regulating functional disorder modules. In addition, the Module_Drug_TargetGene regulatory network for target genes and drugs with potential effect on propofol anesthesia surgery was obtained (Schedule 4-1, Figure 5(b)).

## 4. Discussion

Coronary vascular disease has become a problem facing the world while common treatment is coronary artery bypass surgery (CABG). In the United States alone, about 500,000 patients need CABG surgery every year. OPCABG is a form of CABG surgery. According to statistics, due to its hemodynamic abnormalities during surgery [19], only about 20% of CABG surgeries are now performed under nonextracorporeal circulation. OPCABG has exhibited some advantages, especially in reducing postoperative complications such as systemic inflammation and myocardial and cerebral damage [20, 21]. Acute kidney injury (AKI) is also one of the common postoperative complications of OPCABG, which may be related to chlorine free radical IVF [22]. We need to anesthetize patients before OPCABG surgery, and anesthesia may also cause unexpected hypothermia, which may be another complication of perioperative cardiovascular [23]. Therefore, it is very important to choose a suitable anesthesia method.

In this study, the typical anesthesia methods were selected: sevoflurane anesthesia and propofol anesthesia. The effects of two anesthesia methods on OPCABG were studied, based on a functional module network. We obtained microarray data from sevoflurane anesthesia (inhalation anesthesia) and propofol anesthesia (intravenous anesthesia) from the GEO database, differentially analysed the two groups of data, respectively, and obtained 5701 and 3210 differentially expressed genes, respectively. We believe that these differential genes are dysfunctional molecules. We then performed WGCNA analysis on the two groups of differential genes, and seven functional barrier modules were obtained by sevoflurane anesthesia clustering, including PDCD6IP, WDR3, and other core genes; propofol anesthesia clustering obtained two functional disorders module including two core genes, KCNB2 and LHX2. After obtaining the dysfunction module, the mechanism of action cannot be fully explained, so further enrichment analysis of the functions and pathways of interest is needed. The GO enrichment analysis of the sevoflurane anesthesia-related module gene found that it mainly focused on biological processes such as anion antiporter activity, and KEGG enrichment analysis found that it was mainly related to the PI3K-Akt signalling pathway. The GO enrichment analysis of the gene related to propofol anesthesia showed that it mainly focused on biological processes such as channel activity. The KEGG enrichment analysis found that it was mainly relative with the neuroactive ligand-receptor interaction plasma pathway. In addition, through the coexpression network, some scholars have found that anesthetics may protect the heart by activating the complement and coagulation system [24]. There is still some controversy about the impact of two anesthesia methods on surgery. On the one hand, clinical data have shown that sevoflurane can reduce death within 180 to 365 days after surgery with positive inotropic and vasoconstrictor support with minimal impact on cardiac index [25]. It can also be labelled with the sensitive biomarkers miR-499 and miR-208b [26]. On the other hand, studies have shown that 30% of hernia in propofol anesthesia improves hemodynamic stability by reducing the patient's norepinephrine requirement [27]. Of course, some new anesthesia methods are now available, such as remifentanil target-controlled infusion, constant-rate infusion, chest epidural anesthesia (EA), and postoperative epidural infusion (EI) [28, 29]. During the operation, drugs are often used, so we analysed the potential drugs of the two anesthesia methods to provide guidance for clinical use. The pivot analysis of potential drug regulators in this study showed that there were 229 potential drugs in the operation of sevoflurane anesthesia and zinc regulated three functional disorder modules through core genes such as AHSG and F12; pivot analysis of potential drug regulators during propofol anesthesia surgery showed 67 potential agents, including propofol, methadone, and buprenorphine which regulate two functional barrier modules via the four genes CYP2C8, OPRM1, CYP2C18, and CYP2C19. This study provides guidance on clinical use or treatment by comparing the effects of two anesthesias on surgery and its potential drugs.

## Figures and Tables

**Table 1:** Key genes of sevoflurane anesthesia related modules.

Colour	Hub genes	Module
Black	PDCD6IP	m6
Blue	DNAH10	m1
Brown	WDR3	m2
Green	PROP1	m5
Red	ASCL2	m7
Turquoise	LRRC2-AS1	m3
Yellow	SDC3	m4

**Table 2:** Key genes of propofol anesthesia related modules.

Colour	Hub genes	Module
Blue	KCNB2	m2
Turquoise	LHX2	m1

## Data Availability

The data associated with this manuscript can be accessed from GSE4386.
